# Blood sampling patterns in primary care change several years before a cancer diagnosis

**DOI:** 10.2340/1651-226X.2024.28559

**Published:** 2024-02-13

**Authors:** Mathilde Egelund Christensen, Mia Klinten Grand, Margit Kriegbaum, Bent Struer Lind, Kirsten Grønbæk, Frederik Persson, Christoffer Johansen, Christen Lykkegaard Andersen

**Affiliations:** a Department of Hematology, Copenhagen University Hospital, Rigshospitalet, Denmark; b Centre for General Practice, Institute for Public Health, University of Copenhagen, Denmark; c Danish Cancer Society, Denmark; d Department of Clinical Biochemistry, Copenhagen University Hospital, Hvidovre, Denmark; e Steno Diabetes Center Copenhagen, Herlev, Denmark; f Department of Clinical Oncology, Copenhagen University Hospital, Rigshospitalet, Denmark

**Keywords:** Primary care, early detection of cancer, neoplasms, epidemiologic studies, public reporting of healthcare data, clinical chemistry tests

## Background

A sudden increase in blood sample requisitions from primary care physicians may indicate an incentive to investigate certain symptoms further. Patients with slowly progressing malignancies may debut with insidious symptoms and seek medical attention with increasing frequency, which indeed has been demonstrated through different markers of primary care activity in the last 1–2 years prior to a cancer diagnosis [1, 2]. However, within hematological malignancies in particular, early signs of slowly progressing or pre-malignant conditions may in fact be detectable several years before the malignant disease is diagnosed [3–5]. Identifying early signs of malignancy or pre-malignancy is important in order to increase chances of successful treatment and lower morbidity and mortality [6–8]. Even in asymptomatic and otherwise low-risk hematological patients, it has been demonstrated that early detection may enable risk stratification for follow-up of the (pre-)malignant conditions, help provide relevant care and improve early disease detection in the right patients [9]. In this descriptive study we aimed to describe the pre-diagnostic activity in primary care going back 15 years prior to the malignant diagnosis in order to explore if laboratory activity may indicate that early (pre-)malignant conditions register in primary care years before a diagnosis is made. This was done by describing the blood sampling activity patterns in primary care in both cancer patients to-be and controls, looking at both solid tumors and hematological malignancies. The overall aim of the study was to evaluate the potential for earlier diagnosis of certain malignant diseases.

## Methods

### Population

Cancer cases consisted of patients in the greater Copenhagen area with a malignant diagnosis registered in the exhaustive nationwide Danish Cancer Registry from 2001 to 2015 [10]. We excluded patients under the age of 16 years at the time of diagnosis and patients with previous cancers. Controls were included from the Population Register including all Danish residents with data available from the beginning of 1976 [11]. Cases and controls were matched 1:10 on sex and year of birth, controls being alive and without a cancer diagnosis and living in the greater Copenhagen area as of January 1st of the year of diagnosis for the case, forming our final study population. Cases could serve as controls before their cancer diagnoses and not all controls were unique, however 98% of patients were matched to 10 unique controls.

### Data

Malignant diagnoses included all cancers namely, solid tumors as well as hematological malignancies, except non-melanoma skin cancer and certain diagnoses considered to be of pre-malignant or unknown character [12, 13]. Cancer diagnoses were grouped according to organ of origin that is, head and neck, digestive system, respiratory system etc. (Supplementary Table 1).

Laboratory data were extracted from the Copenhagen Primary Care Laboratory (CopLab) Database encompassing the numerical results of all laboratory work-up ordered by primary care physicians and practicing specialists in the greater Copenhagen area (constituting 20% of the Danish population) from 2000 to 2015, *n* = 112 million results [14]. Nomenclature for properties and units (NPU) codes were selected per unique request and test group including only tests requisitioned on the study population prior to the diagnosis of the case. We excluded test results from the last month leading up to the malignant diagnosis in order to avoid tests possibly relating to diagnostic processes in secondary care. For the purpose of this study, we included only the number of tests performed and not the numerical results of these specific tests.

### Statistics

We quantified the number of incident cancer cases within study period as well as per calendar year for men and women separately. In addition, we quantified the number of cancer cases during the study period within each organ system by age at disease onset and sex.

The mean number of tests per person years at risk for cancer cases and their matched controls were reported by year going back a maximum of 15 years prior to the malignant diagnosis for the whole population as well as for each cancer type that is, by organ system (Supplementary Table 1). For the last 6 years prior to diagnosis, we plotted the NPU codes requested for the cases relative to the controls year by year to visualize which samples drove any potential increase in blood sample frequency with impending malignancy. The mean number of tests per person years at risk was calculated as the mean number of tests divided by the number of person-years at risk of having a test performed since not everyone was at risk of having a test in the CopLab database throughout the 15 years period that is, not having lived in the greater Copenhagen area throughout the entire period. As this was a descriptive study on observational data, no statistical tests were applied to assess the significance of mean differences, instead we chose to report and discuss the observations made in the next section [15, 16].

## Results

**Figure 1:**
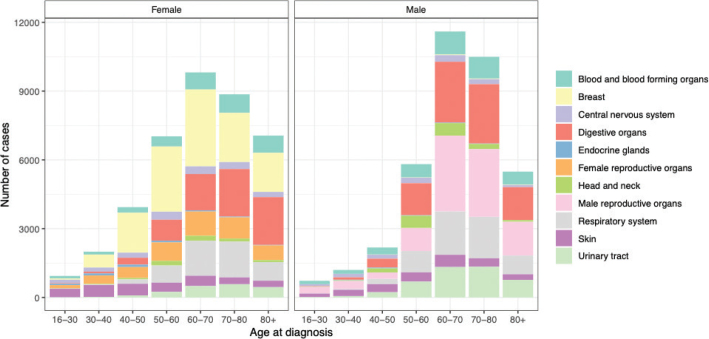
Cancer incidence by age at disease onset and sex.

We ultimately included 77,146 patients with primary malignant disease with a median age of 67 years (interquartile range [57;76]) (Table 1). The overall cancer incidence across age groups was comparable for men and women, but the age distribution differed among the sexes with a larger proportion of women among the 30–50-year-old cancer patients, whereas the majority of the 60–80-year-old cancer patients were men (Figure 1). The most frequent malignant diagnoses were prostate cancer for men and breast cancer for women (Figure 1, Supplementary Table 2). Malignant diagnoses were equally distributed across calendar years – although we observed a small peak in incident malignant diagnoses for women in 2008–2009 aligning with the introduction of a nationwide screening program for breast cancer (Supplementary Figure 1).

**Table 1:** Characteristics of the study population.

	Cases		Controls	
	Female	Male	Female	Male
	*n* = 39,641	*n* = 37,505	*n* = 396,410	*n* = 375,050
Age, years	66 [55;77]^a^	68 [59;76]	66 [55;77]	68 [59;76]
Year^b^, *n* (%)				
2001–2005	12,739 (32.1)	11,603 (30.9)	127,390 (32.1)	116,030 (30.9)
2006–2010	13,398 (33.8)	12,848 (34.3)	133,980 (33.8)	128,480 (34.3)
2011–2015	13,504 (34.1)	13,054 (34.8)	135,040 (34.1)	130,540 (34.8)
Cohabitation status, *n* (%)				
Cohabiting	17,982 (45.4)	23,500 (62.7)	186,184 (47.0)	238,859 (63.7)
Living alone	21,659 (54.6)	14,005 (37.3)	210,226 (53.0)	136,191 (36.3)
Immigration status, *n* (%)				
Danish	36,424 (91.9)	34,290 (91.4)	351,773 (88.7)	333,632 (89.0)
Immigrant	3,065 (7.7)	3,081 (8.2)	42,981 (10.8)	39,998 (10.7)
Descendant^c^	152 (0.4)	134 (0.4)	1,656 (0.4)	1,420 (0.4)
Educational level^d^, *n* (%)				
Low	13,336 (36,8)	10,526 (30.2)	126,264 (35.3)	98,141 (28.3)
Medium	13,623 (37.6)	15,793 (45.4)	134,486 (37.6)	153,743 (44.4)
High	9,237 (25.5)	8,492 (24.4)	97,247 (27.2)	94,437 (27.3)
Unavailable	3,445	2694	38,413	28,729

a Median [IQR], all such numbers;

b Year of diagnosis for the case;

c Descendant of immigrant;

d Educational level was defined according to the International Standard Classification of Education (ISCED): Low: ISCED 0–2 (primary school and high school); Medium: ISCED 3–4 (secondary education); High: ISCED 5–6 (post-secondary education).

**Figure 2:**
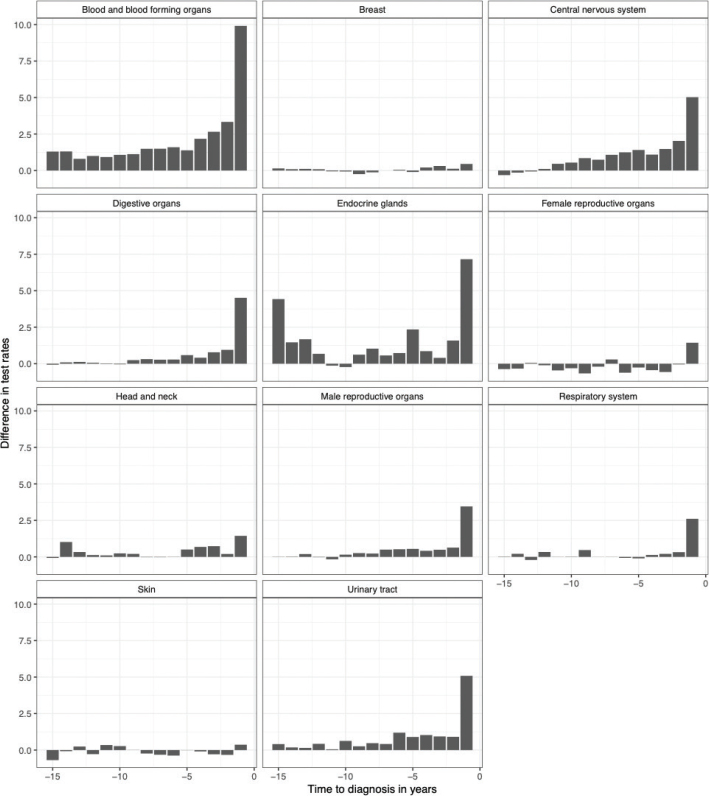
Difference in rate of tests between cases and controls 15 years prior to the diagnosis according to cancer group.

For most malignancies the mean number of tests per person year at risk in primary care increased within the last year before the cancer diagnosis, with cases having up to 10 additional tests performed per year at risk, relative to the controls (Figure 2). This, however, was not the case for malignant melanoma and breast cancer, where test frequencies appeared similar for cases and controls throughout the follow-up period. In the case of CNS (central nervous system) tumors and hematological malignancies we observed an increase in blood sampling activity several years (>5) before the diagnosis driven by observations in myelodysplastic syndromes and the benign CNS tumors (Supplementary Table 3, Supplementary Figure 2). For endocrine malignancies that is, thyroid cancer a two-peaked pattern was observed with a relatively high number of tests 15 years before diagnosis and again in the last year leading up to the diagnosis.

We then analyzed which type of blood samples drove the increased activity among general practitioners on cancer cases-to-be and found that these were largely consisting of standard tests such as complete blood cell counts and kidney function tests. For thyroid cancer, thyroid stimulating hormone (TSH) had the highest rate difference compared with controls throughout the follow-up period.

## Discussion

### Main findings

This is a descriptive study exploring the number of blood tests performed in cancer patients and healthy controls up to 15 years prior to the malignant diagnosis. We identify prostate cancer as the most frequent cancer type for men and breast cancer for women in concurrence with the literature [17]. We also demonstrate that the frequency of blood tests performed on primary care patients increases within 1 year leading up to most malignant diagnoses, however, hematological malignancies and CNS tumors showed an increased sampling activity as much as 5 years before the diagnosis indicating a potential window for earlier diagnosis in these cases.

### Comparison with the literature

Blood sampling frequency and other measures of primary care activity have been described in specific cancer diagnoses before showing increased activity 1–2 years before the diagnosis [1, 18]. This study, however, shows a considerably longer period with increased laboratory work-up for hematological patients and CNS tumors going back more than 5 years prior to the diagnosis. The +5-year latency period in hematological cancers is driven mainly by the myelodysplastic syndromes, which tend to debut with subtle changes in blood hematology, the significance of which can be unclear until the condition develops into more fulminant disease [3, 19]. In some instances the increased blood sample frequency could also reflect the follow-up of comorbidities for example, certain autoimmune diseases with a known association with secondary hematological malignancies, although these secondary malignancies are rare compared with the sporadic cases [20–22].

We observed a similar prolonged period of +5 years with increased attention in primary care before a CNS tumor diagnosis driven by the secreting pituitary adenomas as well as the more slowly developing benign CNS tumors. The endocrine tumors of the brain can be difficult to diagnose and benign CNS tumors may also debut with unspecific symptoms such as dementia possibly prolonging the period from symptom onset to diagnosis [23–25]. In thyroid cancer we noticed an interesting two-peak pattern with sample frequency increasing at -15 years and -1 year before diagnosis, albeit numbers were small. These two peaks were largely comprised by TSH measurements and could indicate a previous benign thyroid disease with chronic inflammation – such as an autoimmune thyroiditis – predating the thyroid cancer by several years [26, 27].

## Strengths and limitations

The observations presented in this study are strengthened by the population-based design and the large sample size. The merging of high-quality nation-wide register data with exhaustive primary care laboratory real-world data provides a unique insight into real-life clinical practice predating a malignant diagnosis. We regard the observation of a small peak in incident malignant diagnoses for women in 2008–2009 aligning with the introduction of a nationwide screening program for breast cancer as a sign of data validity. Such validity we believe is further indicated by the lack of increased blood sampling activity before a breast cancer or malignant melanoma diagnosis, since suspicion of these tumors are based on clinical examinations and the patient is referred to a specialized hospital unit usually without delaying with laboratory work-up in primary care. A major limitation to our design is the lack of clinical data from primary care physicians including medical history and the indications and symptoms leading to the blood sample requisition. Cancer patients are known to be more comorbid than their cancer-free peers [28] and the lack of clinical information in our study makes it difficult to separate investigations of possible malignancy from routine follow-up of chronic illnesses. However, if comorbidities were the primary drivers of the observations made we would expect to see the longest pre-diagnostic flair in sample activity in for example, pulmonary cancers or colorectal cancers more thoroughly linked to comorbidities and lifestyle factors than is the case for hematological cancers and CNS tumors [29–34], making it more likely that the blood sampling patterns identified in hematological cancers and CNS tumors could in fact reflect the early stages of development of these diseases. Studies of real-world data are subjected to some level of surveillance bias, individuals with increased health-seeking behavior being more likely to have a slowly progressing malignancy detected. However, the spiked sampling activity observed in myelodysplastic syndrome was not observed to the same extent in chronic lymphocytic leukemia or plasma cell diseases although these are just as often slowly progressing and more frequently occurring [35, 36]. We therefore deem it unlikely that surveillance bias alone drove the observations presented in this study.

This study neither enables us to assess the predictive property of each individual blood test nor does it take the numerical results of the tests into account, which means that we are not able to conclude on the prognostic or diagnostic value of the specific tests in the pre-diagnostic period.

## Implications and conclusion

In this descriptive study we confirm previous observations of increasing primary care activity within the last 1–2 years leading up to most malignant diagnoses. In addition, we demonstrate a >5-year period of increased activity for benign CNS tumors and certain hematological diseases, most remarkedly myelodysplastic syndromes, which may be indicative of a potential window characterized by early manifestations of these diseases. Further studies in the pre-diagnostic period of benign CNS tumors and certain hematological cancers may be warranted.

## Supplementary Material

Blood sampling patterns in primary care change several years before a cancer diagnosis

Blood sampling patterns in primary care change several years before a cancer diagnosis

## Data Availability

The Copenhagen Primary Care Laboratory Database (CopLab) is administered by Department of Public Health, University of Copenhagen and access to data is governed by the CopLab steering group. Data cannot be made publicly available as it contains personal information. However, researchers irrespective of nationality and affiliation may submit applications, which will then be assessed by the Steering Group of CopLab for data access. https://publichealth.ku.dk/research/databases-for-collaboration/coplab/
