# Supplementary figures and images for: Blood sampling patterns in primary care change several years before a cancer diagnosis
Source: Acta Oncol. 2024 Feb 13;63:28559. doi: 10.2340/1651-226X.2024.28559 (PMC11332553; doi:10.2340/1651-226X.2024.28559)

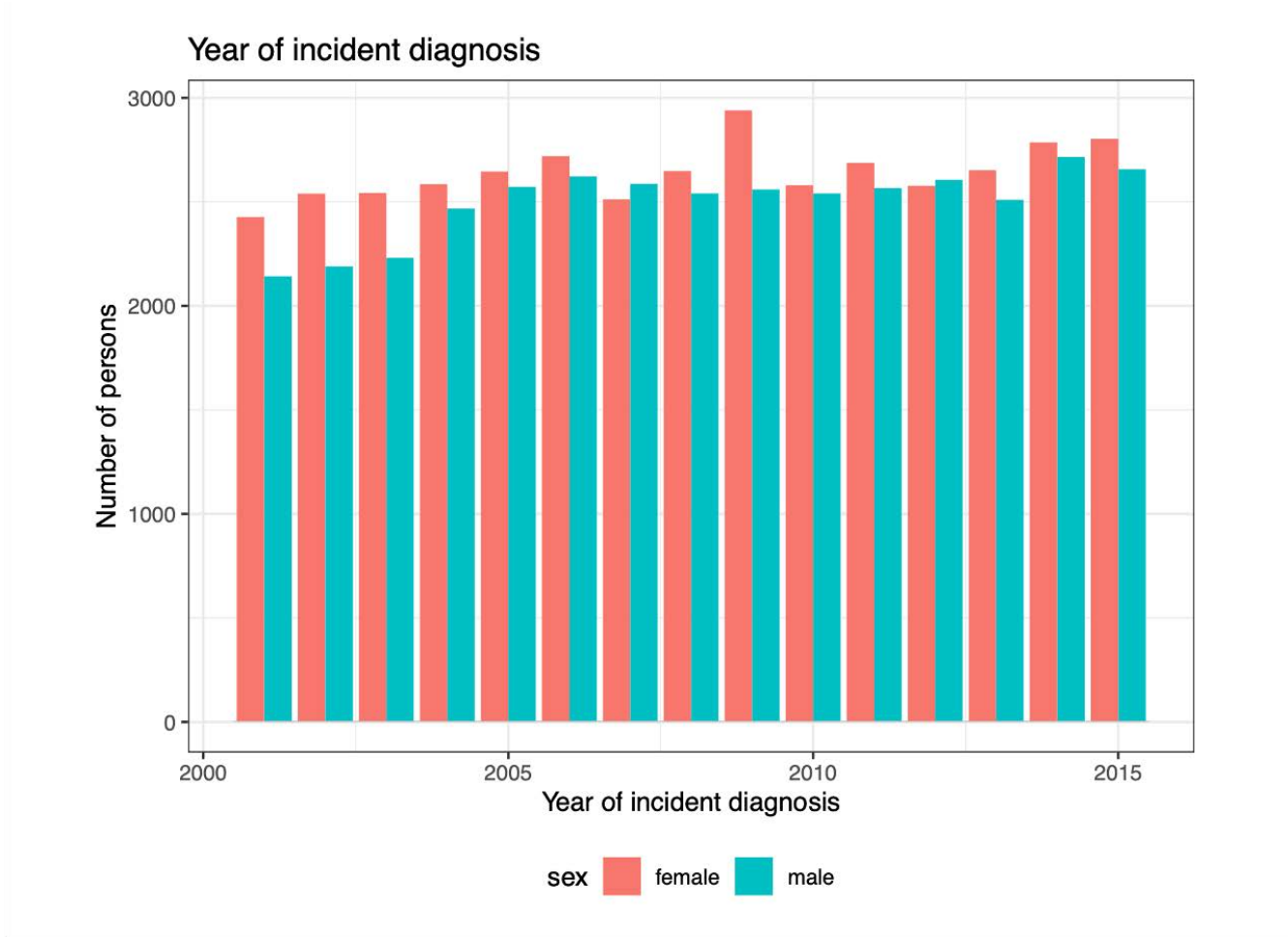

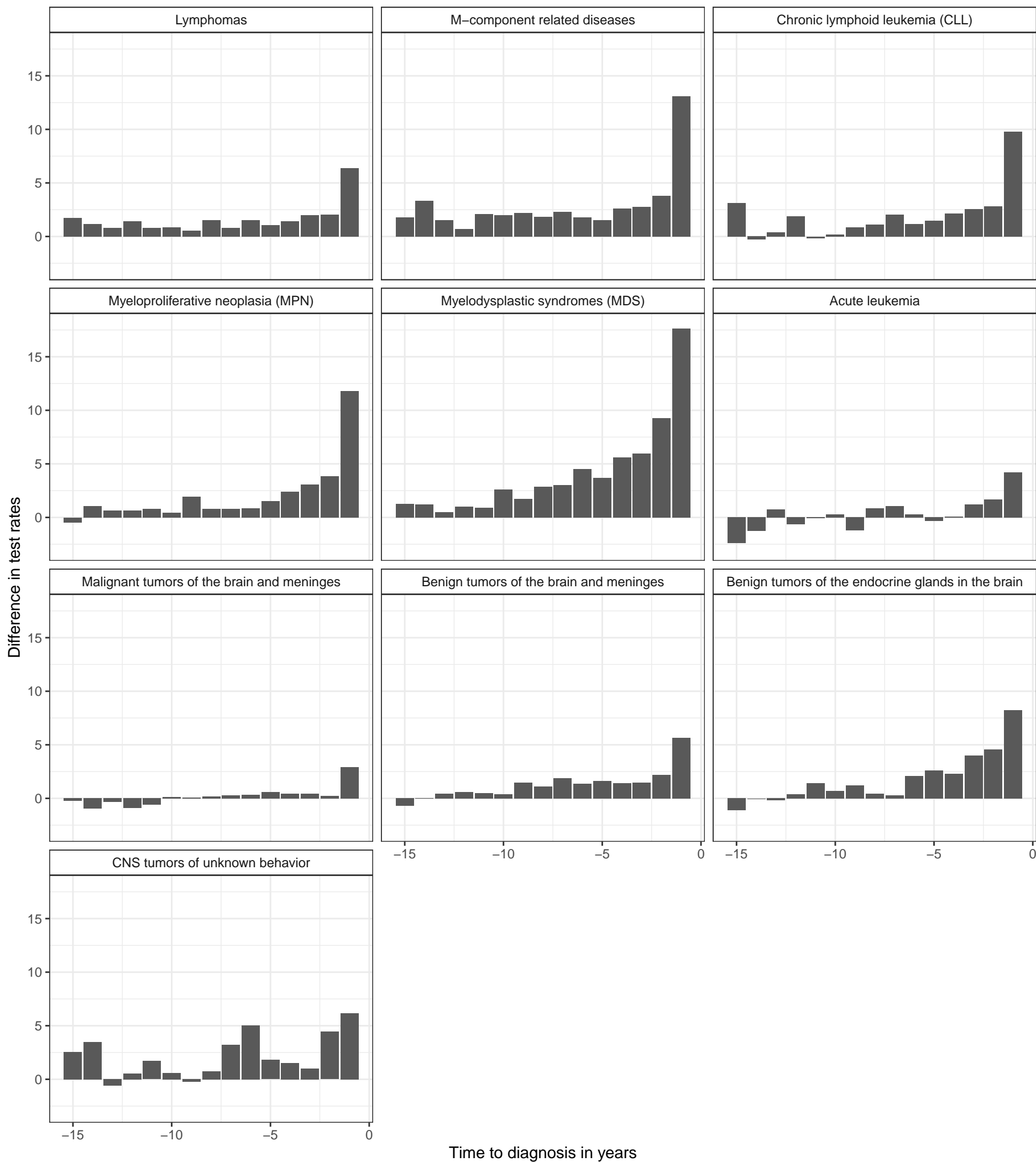

Supplement: Blood sampling patterns in primary care change several years before a cancer diagnosis [file AO-63-28559-s1.pdf]
