# Supplementary material for: Blood sampling patterns in primary care change several years before a cancer diagnosis
Source: Acta Oncol. 2024 Feb 13;63:28559. doi: 10.2340/1651-226X.2024.28559 (PMC11332553; doi:10.2340/1651-226X.2024.28559)
Supplement: Blood sampling patterns in primary care change several years before a cancer diagnosis [file AO-63-28559-s2.pdf]

Supplementary material has been published as submitted. It has not been copyedited or typeset by Acta Oncologica.

Supplementary Table 1: Defining cancer groups

Organ group	Malignancies	ICD-10 included	ICD-10 excluded[1]
Head and neck	Cancers of the lip, tongue, mouth, salivary gland and pharynx	C00-C14, C46.2	-
Digestive organs	Cancers of the esophagus and stomach, colorectal cancer and cancer of the liver and pancreas	C15-C16, C18-C22, C25	C17, C23-C24, C26
Respiratory system	Cancers of the nasal cavity, middle ear and sinuses, laryngeal cancer and cancer of lung, bronchus, trachea and pleura.	C30-C34, C39, C38.4, C45.0	C37, C38.0-C38.3, C38.8
Bones and joints	Sarcomas	-	C40, C41
Skin	Malignant melanoma	C43	C44, C46.0
Connective tissues	Cancers of mesothelium, peripheral nerves and the peritoneum and retroperitoneum	-	C45.1-C45.9, C46.1, C46.3, C46.7-C46.9, C47, C48, B21.0
Breast	Breast cancer	C50	-
Female reproductive organs	Cancers of the cervix and corpus uteri, ovarian cancer and cancers of the fallopian tube and broad ligament	C53-C56, C57.0, C57.4, C58	C51-C52, C57.7-C57.9
Male reproductive organs	Prostate and testis cancers	C61-C62	C60, C63
Urinary tract	Cancers of kidney, renal pelvis and ureter. Bladder cancer.	C64-C67, D09.0, D30.1-D30.3, D41.1-D41.2, D41.4,	C68, D09.1, D30.4-D30.9, D41.3, D41.7-D41.9
Central nervous system	Cancers of brain and meninges	C70-C71, C75.1-C75.3, D32, D33.0-D33.2, D35.2-D35.4, D42, D43.0-D43.2, D44.3-D44.5	C69, C72, D33.3-D33.9, D43.3-D43.9
Endocrine glands	Thyroid cancer	C73	C74, C75.0, C75.4-C75.9
Blood and blood forming organs	Hodgkin lymphoma, non-Hodgkin lymphoma, multiple myeloma, lymphatic leukemias, myeloproliferative disease, myelodysplastic syndromes, monocytic leukemias, acute myeloid leukemia	C81-C86, C88.0-C88.9, C90-C91, C92.0-C92-8, C93-C96, D45-D46, D47.0-D47.5	C95, C96
Other	Ill-defined and unspecified cancers	-	C76-C80

Organ group	ICD-10	N (%)
Head and neck	C00 Malignant neoplasm of lip	61 (2.7)
	C01 Malignant neoplasm of base of tongue	140 (6.2)
	C02 Malignant neoplasm of other or unspecified part of tongue	277 (12.3)
	C03 Malignant neoplasm of gum	58 (2.6)
	C04 Malignant neoplasm of floor of mouth	133 (5.9)
	C05 Malignant neoplasm of palate	79 (3.5)
	C06 Malignant neoplasm of other or unspecified part of mouth	268 (11.9)
	C07 Malignant neoplasm of parotid gland	100 (4.5)
	C08 Malignant neoplasm of other or unspecified major salivary gland	27 (1.2)
	C09 Malignant neoplasm of tonsil	492 (21.9)
	C10 Malignant neoplasm of oropharynx	267 (11.9)
	C11 Malignant neoplasm of nasopharynx	69 (3.1)
	C12 Malignant neoplasm of piriform sinus	52 (2.3)
	C13 Malignant neoplasm of hypopharynx	222 (9.9)
	C14 Malignant neoplasm of other or ill-defined sites	SN
	C46.2 Kaposi sarcoma of palate	SN
Digestive organs	C15 Malignant neoplasm of oesophagus	1,179 (9.6)
	C16 Malignant neoplasm of stomach	1,228 (10.0)
	C18 Malignant neoplasm of colon	6,213 (50.4)
	C19 Malignant neoplasm of rectosigmoid junction	117 (0.9)
	C20 Malignant neoplasm of rectum	3,186 (25.8)
	C21 Malignant neoplasm of anus and anal canal	367 (3.0)
	C22 Malignant neoplasm of liver and intrahepatic bile ducts	1,066 (8.6)
	C25 Malignant neoplasm of pancreas	2,272 (18.4)
Respiratory system	C30 Malignant neoplasm of nasal cavity and middle ear	112 (1.1)
	C31 Malignant neoplasm of accessory sinuses	40 (0.4)
	C32 Malignant neoplasm of larynx	651 (6.1)
	C33 Malignant neoplasm of trachea	10 (0.09)
	C34 Malignant neoplasm of bronchus and lung	9,571 (90.4)
	C38.4 Malignant neoplasm of pleura	53 (0.5)
	C39 Malignant neoplasm of other and ill-defined sites in the respiratory or intrathoracic organs	7 (0.07)
	C45.0 Mesothelioma of pleura	147 (1.4)
Bones and joints	-	-
Skin	C43 Malignant melanoma of skin	5,154 (100)
Connective tissues	-	-
Breast	C50 Malignant neoplasm of breast	12,478 (100)
Female reproductive organs	C53 Malignant neoplasm of cervix uteri	1,207 (27.4)
	C54 Malignant neoplasm of corpus uteri	1,779 (40.3)
	C55 Malignant neoplasm of uterus, part unspecified	81 (1.8)
	C56 Malignant neoplasm of ovary	1,310 (29.7)
	C57.0 Malignant neoplasm of the fallopian tube	32 (0.7)
	C57.4 Malignant neoplasm of the uterine adnexa	SN
	C58 Malignant neoplasm of placenta	3 (0.07)
Male reproductive organs	C61 Malignant neoplasm of prostate	8,693 (99.7)
	C62 Malignant neoplasm of testis	12 (0.3)
Urinary tract	C64 Malignant neoplasm of kidney except renal pelvis	1,518 (24.0)
	C65 Malignant neoplasm of renal pelvis	158 (2.5)
	C66 Malignant neoplasm of ureter	45 (0.7)
	C67 Malignant neoplasm of bladder	1,981 (31.3)
	D09.0 Carcinoma in situ of bladder	1,067 (16.9)
	D30.1 Benign neoplasm of renal pelvis	33 (0.5)
	D30.2 Benign neoplasm of ureter	17 (0.27)
	D30.3 Benign neoplasm of bladder	854 (13.5)

	D41.1 Neoplasms of uncertain or unknown behavior: renal pelvis	50 (0.8)
	D41.2 Neoplasms of uncertain or unknown behavior: ureter	15 (0.2)
	D41.4 Neoplasms of uncertain or unknown behavior: bladder	582 (9.2)
Central nervous system	C70 Malignant neoplasm of meninges	7 (0.2)
	C71 Malignant neoplasm of brain	1,140 (37.5)
	C75.1 Malignant neoplasm of the pituitary gland	3 (0.1)
	C75.2 Malignant neoplasm of the craniopharyngeal duct	SN
	C75.3 Malignant neoplasm of the pineal gland	SN
	D32 Benign neoplasms of meninges	1,049 (34.5)
	D33.0 Benign neoplasm: brain, supratentorial	101 (3.3)
	D33.1 Benign neoplasm: brain, infratentorial	26 (0.9)
	D33.2 Benign neoplasm: brain, unspecified	58 (1.9)
	D35.2 Benign neoplasm: pituitary gland	411 (13.5)
	D35.3 Benign neoplasm: craniopharyngeal duct	4 (0.1)
	D35.4 Benign neoplasm: pineal gland	19 (0.6)
	D42 Neoplasms of uncertain or unknown behavior: meninges	21 (0.6)
	D43.0 Neoplasms of uncertain or unknown behavior: brain, supratentorial	80 (2.6)
	D43.1 Neoplasms of uncertain or unknown behavior: brain, infratentorial	35 (1.2)
	D43.2 Neoplasms of uncertain or unknown behavior: brain, unspecified	56 (1.8)
	D44.3 Neoplasms of uncertain or unknown behavior: pituitary gland	9 (0.3)
	D44.4 Neoplasms of uncertain or unknown behavior: craniopharyngeal duct	20 (0.7)
	D44.5 Neoplasms of uncertain or unknown behavior: pineal gland	4 (0.1)
Endocrine glands	C73 Malignant neoplasm of thyroid gland	578 (100)
Blood and blood forming organs	C81 Hodgkin lymphoma	310 (4.6)
	C82 Follicular lymphoma	296 (4.3)
	C83 Non-follicular lymphoma	807 (11.9)
	C84 Mature T/NK-cell lymphoma	175 (2.6)
	C85 Other and unspecified types of non-Hodgkin lymphoma	659 (9.7)
	C86 Other specified types of T/NK-cell lymphoma	SN
	C88 Malignant immunoproliferative disease	131 (1.9)
	C90 Multiple myeloma and malignant plasma cell neoplasms	722 (10.6)
	C91 Lymphoid leukemia	1,117 (16.4)
	C92.0 Acute myeloblastic leukemia	317 (4.7)
	C92.1 Chronic myeloid leukemia	143 (2.1)
	C92.2 Atypical chronic myeloid leukemia	8 (0.1)
	C92.3 Myeloid sarcoma	5 (0.07)
	C92.4 Acute promyelocytic leukemia	22 (0.4)
	C92.5 Acute myelomonocytic leukemia	25 (0.3)
	C92.6 Acute myeloid leukemia with 11q23- abnormality	SN
	C92.7 Other myeloid leukemia	14 (0.02)
	C92.8 Acute myeloid leukemia with multilineage dysplasia	5 (0.07)
	C93 Monocytic leukemia	62 (0.9)
	C94 Other leukemias of unspecified cell type	26 (0.4)
	C95 Leukemia of unspecified cell type	SN
	C96 Other and unspecified malignant neoplasms of lymphoid, hematopoietic and related tissue	SN
	D45 Polycythemia vera	348 (5.1)
	D46 Myelodysplastic syndromes	514 (7.5)
	D47.0 Histocytic and mast cell tumors of uncertain and uknown behavior	17 (0.2)
	D47.1 Chronic myeloproliferative disease	239 (3.5)
	D47.2 Monoclonal gammopathy of undetermined potential	540 (7.9)

	D47.3 Essential thrombocythemia	390 (5.7)
	D47.4 Osteomyelofibrosis	39 (0.6)
	D47.5 Chronic eosinophilic leukemia	10 (0.1)
Other	-	

Supplementary Table 2: Grouping of hematological malignancies and tumors of the central nervous system.

	Hematological malignancies
**Disease group**	**ICD-10 diagnosis[1]**
Lymphomas	C81 Hodgkin lymphoma
	C82 Follicular lymphoma
	C83 Non-follicular lymphoma
	C84 Mature T/NK-cell lymphomas
	C85 Other and unspecified types of non-Hodgkin lymphoma
	C88.3 Immunoproliferative small intestinal disease
	C88.4 Extranodal marginal zone B-cell lymphoma of mucosa associated lymphoid tissue (MALT-lymphoma)
	C88.7 Other malignant immunoproliferative diseases
	C88.9 Malignant immunoproliferative disease, unspecified
	C91.5 Adult T-cell lymphoma/leukemia [HTLV-1-associated]
M-component related diseases	C88.0 Waldenströms macroglobulinemia
	C88.2 Other heavy chain disease
	C90 Multiple myeloma and malignant plasma cell neoplasms
	D47.2 Monoclonal gammopathy of undetermined potential (MGUS)
Chronic lymphoid leukemia (CLL)	C91.1 Chronic lymphoid leukemia of B-cell type
	C91.3 Prolymphocytic leukemia of B-cell type
	C91.4 Hairy cell leukemia
	C91.6 Prolymphocytic leukemia of T-cell type
	C91.7 Other lymphoid leukemia
	C91.9 Lymphoid leukemia, unspecified
	C95.1 Chronic leukemia of unspecified cell type
Myeloproliferative neoplasia (MPN)	C92.1 Chronic myeloid leukemia, BCR/ABL-positive
	C92.2 Atypical chronic myeloid leukemia, BCR/ABL-negative
	D45 Polycythemia Vera
	D47.0 Histiocytic and mast cell tumors of uncertain or unknown behavior
	D47.1 Chronic myeloproliferative disease
	D47.3 Essential thrombocythemia
	D47.4 Osteomyelofibrosis
	D47.5 Chronic eosinophilic leukemia
Myelodysplastic syndromes (MDS)	D46 Myelodysplastic syndromes
	C93.1 Chronic myelomonocytic leukemia
	C93.2 Juvenile myelomonocytic leukemia
	C93.9 Monocytic leukemia, unspecified
Acute leukemia	C91.0 Acute lymphoblastic leukemia
	C91.8 Mature B-cell leukemia, Burkitt type
	C92.0 Acute myeloblastic leukemia
	C92.3 Myeloid sarcoma
	C92.4 Acute promyelocytic leukemia
	C92.5 Acute myelomonocytic leukemia
	C92.6 Acute leukemia with 11q23-abnormality
	C92.7 Other myeloid leukemia
	C92.8 Acute myeloid leukemia with multilineage dysplasia

	C93.0 Acute monoblastic/monocytic leukemia<br>C94.0 Acute erythroid leukemia<br>C94.2 Acute megakaryoblastic leukemia<br>C94.3 Mast cell leukemia<br>C94.4 Acute panmyelosis with myelofibrosis<br>C94.6 Myelodysplastic and myeloproliferative disease, not elsewhere specified<br>C94.7 Other specified leukemias<br>C95.0 Acute leukemia of unspecified cell type<br>C95.7 Other leukemia of unspecified cell type<br>C95.9 Leukemia, unspecified<br>C96 Other and unspecified malignant neoplasms

	**Tumors of the Central Nervous System**
**Disease group**	**ICD-10 diagnosis**[1]
Malignant tumors of the brain and meninges	C70 Malignant neoplasm of meninges<br>C71 Malignant neoplasm of brain<br>C75.1 Malignant neoplasm: pituitary gland<br>C75.2 Malignant neoplasm: craniopharyngeal duct<br>C75.3 Malignant neoplasm: pineal duct
Benign tumors of the brain and meninges	D32.0 Benign neoplasm: cerebral meninges<br>D33.0 Benign neoplasm: brain, supratentorial<br>D33.1 Benign neoplasm: brain, infratentorial<br>D33.2 Benign neoplasm: brain, unspecified
Benign tumors of the endocrine glands in the brain	D35.2 Benign neoplasm: pituitary gland<br>D35.3 Benign neoplasm: craniopharyngeal duct<br>D35.4 Benign neoplasm: pineal gland
CNS tumors of unknown behavior	D42 Neoplasms of uncertain or unknown behavior: cerebral meninges<br>D43.0 Neoplasms of uncertain or unknown behavior: brain, supratentorial<br>D43.1 Neoplasms of uncertain or unknown behavior: brain, infratentorial<br>D43.2 Neoplasms of uncertain or unknown behavior: brain, unspecified<br>D44.3 Neoplasms of uncertain or unknown behavior: pituitary gland<br>D44.4 Neoplasms of uncertain or unknown behavior: craniopharyngeal duct<br>D44.5 Neoplasms of uncertain or unknown behavior: pineal gland
